# 8-Hy­droxy-5-(hy­droxy­meth­yl)quinolin-1-ium chloride

**DOI:** 10.1107/S1600536812024233

**Published:** 2012-06-02

**Authors:** Majda Fathi, Youssef Fouham, El Hassan Arbib, Brahim Lakhrissi, Mohamed Saadi, Lahcen El Ammari

**Affiliations:** a Laboratoire de Physico-chimie des Matériaux Vitreux et Cristallisés, Equipe de Physico-chimie des Matériaux Inorganiques, Faculté des Sciences, Université Ibn Tofail, Kénitra, Morocco; b Laboratoire d’Agroressources et Génie des Procédés, Faculté des Sciences, Université Ibn Tofail Kénitra, Morocco; c Laboratoire de Chimie du Solide Appliquée, Faculté des Sciences, Université Mohammed V-Agdal, Avenue Ibn Battouta, BP 1014, Rabat, Morocco

## Abstract

The title compound, C_10_H_10_NO_2_^+^·Cl^−^, contains a quinoline ring system which is essentially planar, with the largest deviation from the mean plane being 0.017 (1) Å. In the crystal, the ion pairs and their inversion-symmetry-related partners are linked by N—H⋯Cl and O—H⋯Cl hydrogen bonds to form tetramers which are further connected through O—H⋯O hydrogen bonds, building infinite one-dimensional chains parallel to the [010] direction.

## Related literature

For anti­oxidant properties, see: Kayyali *et al.* (1998 ▶). For the synthesis of some substituted 8-quinolinol derivatives, see: Mishra *et al.* (2004 ▶). For the application of the corresponding aluminium complexes, see: Tang *et al.* (1989 ▶); Chen & Shi (1998 ▶); Shougen *et al.* (2000 ▶). For application as a promising display, see: Cao *et al.* (1996 ▶); Wu *et al.* (2003 ▶). For the synthesis, see: Zheng *et al.* (2005 ▶). 
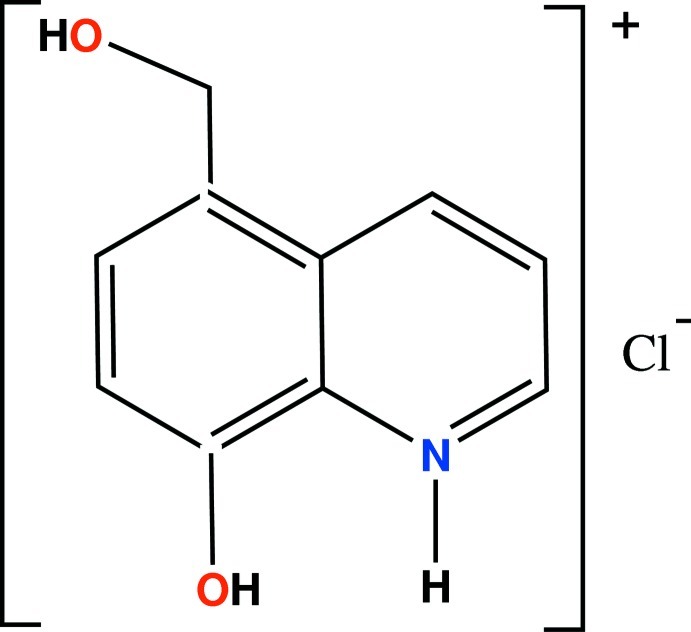


## Experimental

### 

#### Crystal data

C_10_H_10_NO_2_^+^·Cl^−^
*M* *_r_* = 211.64
Monoclinic, 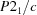

*a* = 6.9081 (5) Å
*b* = 8.0577 (5) Å
*c* = 17.1890 (11) Å
β = 101.183 (3)°
*V* = 938.63 (11) Å^3^
*Z* = 4
Mo *K*α radiation
μ = 0.38 mm^−1^
*T* = 296 K
0.54 × 0.43 × 0.12 mm

#### Data collection

Bruker X8 APEX diffractometer
22727 measured reflections
4615 independent reflections
3679 reflections with *I* > 2σ(*I*)
*R*_int_ = 0.024

#### Refinement

*R*[*F* ^2^ > 2σ(*F* ^2^)] = 0.039
*wR*(*F* ^2^) = 0.124
*S* = 1.07
4615 reflections
127 parameters
H-atom parameters constrained
Δρ_max_ = 0.50 e Å^−3^
Δρ_min_ = −0.20 e Å^−3^

### 

Data collection: *APEX2* (Bruker, 2005 ▶); cell refinement: *APEX2*; data reduction: *SAINT* (Bruker, 2005 ▶); program(s) used to solve structure: *SHELXS97* (Sheldrick, 2008 ▶); program(s) used to refine structure: *SHELXL97* (Sheldrick, 2008 ▶); molecular graphics: *ORTEP-3 for Windows* (Farrugia, 1997 ▶); software used to prepare material for publication: *PLATON* (Spek, 2009 ▶) and *publCIF* (Westrip, 2010 ▶).

## Supplementary Material

Crystal structure: contains datablock(s) I, global. DOI: 10.1107/S1600536812024233/fj2562sup1.cif

Structure factors: contains datablock(s) I. DOI: 10.1107/S1600536812024233/fj2562Isup2.hkl

Supplementary material file. DOI: 10.1107/S1600536812024233/fj2562Isup3.cml

Additional supplementary materials:  crystallographic information; 3D view; checkCIF report

## Figures and Tables

**Table 1:** Hydrogen-bond geometry (Å, °)

*D*—H⋯*A*	*D*—H	H⋯*A*	*D*⋯*A*	*D*—H⋯*A*
N1—H1*N*⋯Cl1	0.86	2.24	3.0261 (8)	152
O1—H1*O*⋯O2^i^	0.82	1.78	2.5841 (10)	166
O2—H2*O*⋯Cl1^ii^	0.82	2.21	3.0281 (8)	172

Symmetry codes: (i) 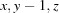; (ii) 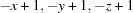.

**Fig. 1.**
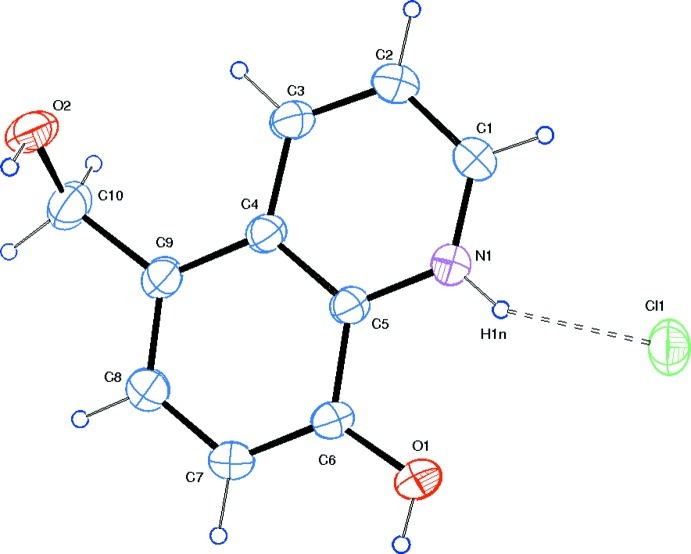
Molecular structure of the title compound with the atom-labelling scheme. Displacement ellipsoids are drawn at the 50% probability level. H atoms are represented as small circles.

**Fig. 2.**
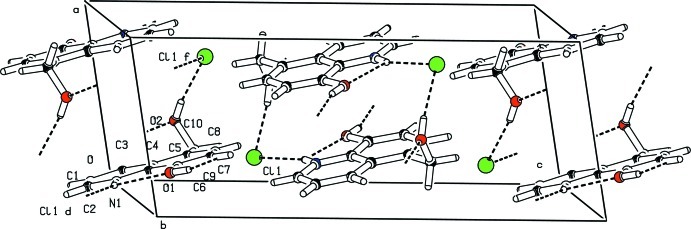
Molecule and its symmetry through the inversion center linked by hydrogen bonds and building dimers.

## supplementary crystallographic information

### Comment

8-Quinolinol is a strong iron chelator with antioxidant property (Kayyali *et al.* 1998). 5-Chloromethyl-8-hydroxyquinoline hydrochloride (I) is used as an intermediate in the synthesis of 5-hydroxymethyl-8-quinolinol and some substituted 8-quinolinol derivatives (Mishra *et al.* (2004). The corresponding aluminium complexes has been used as an excellent Organic Light-Emitting Diodes (OLEDs) (Tang *et al.* (1989), Chen & Shi (1998), Shougen *et al.* (2000)) witch are currently under intensive investigation for application as a promising display technology due to their high luminous efficiency and capability of emitting full colour flat displays (Cao *et al.* (1996), Wu *et al.* (2003)). The present work describes the crystal structure of C_10_H_10_NO_2_.Cl (scheme 1) obtained from the X-ray diffraction data on single-crystal.

The 5-(hydroxymethyl)-8-quinolinol hydrochloride molecule structure is built up from two fused six-membered rings linked to CH_2_OH and to OH groups as shown in Fg.1. The fused-ring system is essentially planar, with the maximum deviation of 0.017 (1) Å from C7 atom. The dihedral angle between them does not exceed 1.15 (5)°. The hydroxide O2—H linked to –C10H_2_– form an angle of 56.68 (6) ° with the mean plane of the quinolin ring. In the crystal, each molecule and its symmetry through the inversion center are linked by N—H···Cl and O—H···Cl hydrogen bonds in the way to form dimers as shown in Fig.2. These dimers are further connected through O—H···O hydrogen bonds building infinite one-dimensional chains parallel to [0 1 0] direction (Table 1).

### Experimental

5-Chloromethyl-8-hydroxyquinoline hydrochloride (I) was synthesized according to the method described by Zheng *et al.* (2005). A mixture of 10.0 g (0.068 mol) of 8-hydroxyquinoline, 11 ml of concentrated hydrochloric acid, and 11 ml (0.397 mol) of 37% formaldehyde was treated with hydrogen chloride gas and stirred for 6 h. The solution was allowed to stand at room temperature for 2 h without stirring. The yellow solid obtained was collected on a filter, washed with acetone or alcohol, and dried under vacuum to give 5-chloromethyl-8-hydroxyquinoline hydrochloride (I) (13.0 g, 98%). The compound obtained was dissolved in distilled water in a box Petrys and let in air at room temperature. After 10 days, transparent single crystals as platelets were isolated. X-ray diffraction analysis shows that the obtained product is the 5-(hydroxymethyl)-8-quinolinol hydrochloride.

### Refinement

H atoms were located in a difference map and treated as riding with N—H = 0.86 Å, C—H = 0.93 Å (aromatic), C—H = 0.97 Å (methylene) and O—H = 0.82 Å with *U*_iso_(H) = 1.2 *U*_eq_ (aromatic, methylene) and *U*_iso_(H)= 1.5 *U*_eq_ (OH).

### Crystal data

**Table:**

C_10_H_10_NO_2_^+^·Cl^−^	*F*(000) = 440
*M**_r_* = 211.64	*D*_x_ = 1.498 Mg m^−^^3^
Monoclinic, *P*2_1_/*c*	Mo *K*α radiation, λ = 0.71073 Å
Hall symbol: -p_2ybc	Cell parameters from 4615 reflections
*a* = 6.9081 (5) Å	θ = 2.8–36.5°
*b* = 8.0577 (5) Å	µ = 0.38 mm^−^^1^
*c* = 17.1890 (11) Å	*T* = 296 K
β = 101.183 (3)°	Needle, colourless
*V* = 938.63 (11) Å^3^	0.54 × 0.43 × 0.12 mm
*Z* = 4	

### Data collection

**Table:**

Bruker X8 APEX diffractometer	3679 reflections with *I* > 2σ(*I*)
Radiation source: fine-focus sealed tube	*R*_int_ = 0.024
Graphite monochromator	θ_max_ = 36.5°, θ_min_ = 2.8°
φ and ω scans	*h* = −11→11
22727 measured reflections	*k* = −12→13
4615 independent reflections	*l* = −28→28

### Refinement

**Table:**

Refinement on *F*^2^	Primary atom site location: structure-invariant direct methods
Least-squares matrix: full	Secondary atom site location: difference Fourier map
*R*[*F*^2^ > 2σ(*F*^2^)] = 0.039	Hydrogen site location: difference Fourier map
*wR*(*F*^2^) = 0.124	H-atom parameters constrained
*S* = 1.07	*w* = 1/[σ^2^(*F*_o_^2^) + (0.0694*P*)^2^ + 0.1142*P*] where *P* = (*F*_o_^2^ + 2*F*_c_^2^)/3
4615 reflections	(Δ/σ)_max_ = 0.001
127 parameters	Δρ_max_ = 0.50 e Å^−^^3^
0 restraints	Δρ_min_ = −0.20 e Å^−^^3^

### Special details

**Table:**

Geometry. All s.u.'s (except the s.u. in the dihedral angle between two l.s. planes) are estimated using the full covariance matrix. The cell s.u.'s are taken into account individually in the estimation of s.u.'s in distances, angles and torsion angles; correlations between s.u.'s in cell parameters are only used when they are defined by crystal symmetry. An approximate (isotropic) treatment of cell s.u.'s is used for estimating s.u.'s involving l.s. planes.
Refinement. Refinement of *F*^2^ against all reflections. The weighted *R*-factor *wR* and goodness of fit *S* are based on *F*^2^, conventional *R*-factors *R* are based on *F*, with *F* set to zero for negative *F*^2^. The threshold expression of *F*^2^ > 2σ(*F*^2^) is used only for calculating *R*-factors(gt) *etc*. and is not relevant to the choice of reflections for refinement. *R*-factors based on *F*^2^ are statistically about twice as large as those based on *F*, and *R*- factors based on all data will be even larger.

### Fractional atomic coordinates and isotropic or equivalent isotropic displacement parameters (Å^2^)

**Table:**

	*x*	*y*	*z*	*U*_iso_*/*U*_eq_	
Cl1	0.16928 (4)	0.17354 (3)	0.310398 (15)	0.03843 (8)	
O1	0.30276 (12)	0.22164 (9)	0.51946 (4)	0.03652 (16)	
H1O	0.3342	0.1525	0.5547	0.055*	
O2	0.44071 (12)	0.98428 (9)	0.61542 (5)	0.03859 (17)	
H2O	0.5509	0.9455	0.6316	0.058*	
N1	0.19725 (11)	0.47152 (9)	0.41916 (4)	0.02749 (14)	
H1N	0.1996	0.3699	0.4041	0.033*	
C1	0.14320 (16)	0.58746 (13)	0.36492 (5)	0.03366 (18)	
H1	0.1075	0.5578	0.3118	0.040*	
C2	0.13937 (16)	0.75383 (12)	0.38682 (6)	0.0362 (2)	
H2	0.1026	0.8355	0.3486	0.043*	
C3	0.19059 (14)	0.79558 (11)	0.46549 (6)	0.03077 (17)	
H3	0.1887	0.9064	0.4804	0.037*	
C4	0.24629 (12)	0.67260 (10)	0.52439 (5)	0.02444 (14)	
C5	0.24952 (12)	0.50630 (10)	0.49814 (5)	0.02361 (14)	
C6	0.30430 (13)	0.37354 (10)	0.55186 (5)	0.02688 (15)	
C7	0.35081 (16)	0.40924 (12)	0.63135 (5)	0.03253 (18)	
H7	0.3846	0.3239	0.6679	0.039*	
C8	0.34767 (16)	0.57403 (12)	0.65797 (5)	0.03347 (18)	
H8	0.3807	0.5948	0.7121	0.040*	
C9	0.29775 (14)	0.70577 (11)	0.60713 (5)	0.02761 (15)	
C10	0.29652 (17)	0.87978 (12)	0.63908 (6)	0.03501 (19)	
H10A	0.1669	0.9279	0.6209	0.042*	
H10B	0.3200	0.8753	0.6965	0.042*	

### Atomic displacement parameters (Å^2^)

**Table:**

	*U*^11^	*U*^22^	*U*^33^	*U*^12^	*U*^13^	*U*^23^
Cl1	0.04035 (14)	0.03721 (14)	0.03423 (12)	0.00236 (9)	−0.00147 (9)	−0.00972 (8)
O1	0.0523 (4)	0.0204 (3)	0.0344 (3)	0.0019 (3)	0.0024 (3)	0.0011 (2)
O2	0.0372 (4)	0.0224 (3)	0.0521 (4)	−0.0014 (2)	−0.0014 (3)	0.0061 (3)
N1	0.0306 (3)	0.0242 (3)	0.0260 (3)	−0.0013 (2)	0.0016 (2)	−0.0007 (2)
C1	0.0395 (5)	0.0317 (4)	0.0265 (4)	−0.0006 (3)	−0.0018 (3)	0.0027 (3)
C2	0.0431 (5)	0.0286 (4)	0.0326 (4)	0.0007 (4)	−0.0034 (4)	0.0066 (3)
C3	0.0334 (4)	0.0218 (3)	0.0342 (4)	0.0001 (3)	−0.0006 (3)	0.0035 (3)
C4	0.0243 (3)	0.0209 (3)	0.0274 (3)	−0.0018 (2)	0.0033 (3)	0.0011 (2)
C5	0.0242 (3)	0.0209 (3)	0.0251 (3)	−0.0015 (2)	0.0034 (2)	0.0013 (2)
C6	0.0304 (4)	0.0207 (3)	0.0289 (3)	−0.0018 (3)	0.0043 (3)	0.0028 (3)
C7	0.0432 (5)	0.0253 (4)	0.0277 (4)	−0.0024 (3)	0.0037 (3)	0.0053 (3)
C8	0.0450 (5)	0.0295 (4)	0.0254 (3)	−0.0046 (4)	0.0057 (3)	0.0009 (3)
C9	0.0317 (4)	0.0232 (3)	0.0280 (3)	−0.0040 (3)	0.0060 (3)	−0.0014 (3)
C10	0.0425 (5)	0.0263 (4)	0.0369 (4)	−0.0026 (3)	0.0094 (4)	−0.0061 (3)

### Geometric parameters (Å, º)

**Table:**

O1—C6	1.3439 (11)	C3—H3	0.9300
O1—H1O	0.8200	C4—C5	1.4155 (11)
O2—C10	1.4225 (13)	C4—C9	1.4229 (12)
O2—H2O	0.8200	C5—C6	1.4158 (11)
N1—C1	1.3215 (12)	C6—C7	1.3721 (13)
N1—C5	1.3644 (11)	C7—C8	1.4059 (14)
N1—H1N	0.8600	C7—H7	0.9300
C1—C2	1.3941 (14)	C8—C9	1.3757 (13)
C1—H1	0.9300	C8—H8	0.9300
C2—C3	1.3718 (14)	C9—C10	1.5065 (12)
C2—H2	0.9300	C10—H10A	0.9700
C3—C4	1.4155 (12)	C10—H10B	0.9700
			
C6—O1—H1O	109.5	C4—C5—C6	121.75 (7)
C10—O2—H2O	109.5	O1—C6—C7	125.81 (8)
C1—N1—C5	122.74 (8)	O1—C6—C5	115.99 (8)
C1—N1—H1N	118.6	C7—C6—C5	118.19 (8)
C5—N1—H1N	118.6	C6—C7—C8	120.44 (8)
N1—C1—C2	120.45 (9)	C6—C7—H7	119.8
N1—C1—H1	119.8	C8—C7—H7	119.8
C2—C1—H1	119.8	C9—C8—C7	122.67 (8)
C3—C2—C1	119.16 (8)	C9—C8—H8	118.7
C3—C2—H2	120.4	C7—C8—H8	118.7
C1—C2—H2	120.4	C8—C9—C4	118.25 (8)
C2—C3—C4	121.05 (8)	C8—C9—C10	120.34 (8)
C2—C3—H3	119.5	C4—C9—C10	121.41 (8)
C4—C3—H3	119.5	O2—C10—C9	113.14 (8)
C3—C4—C5	116.98 (8)	O2—C10—H10A	109.0
C3—C4—C9	124.33 (8)	C9—C10—H10A	109.0
C5—C4—C9	118.69 (7)	O2—C10—H10B	109.0
N1—C5—C4	119.60 (7)	C9—C10—H10B	109.0
N1—C5—C6	118.65 (7)	H10A—C10—H10B	107.8

### Hydrogen-bond geometry (Å, º)

**Table:**

*D*—H···*A*	*D*—H	H···*A*	*D*···*A*	*D*—H···*A*
N1—H1*N*···Cl1	0.86	2.24	3.0261 (8)	152
O1—H1*O*···O2^i^	0.82	1.78	2.5841 (10)	166
O2—H2*O*···Cl1^ii^	0.82	2.21	3.0281 (8)	172

Symmetry codes: (i) *x*, *y*−1, *z*; (ii) −*x*+1, −*y*+1, −*z*+1.
